# Disparate Changes in Plasma and Brainstem Cytokine Levels in Adult and Ageing Rats Associated with Age-Related Changes in Facial Motor Neuron Number, Snout Muscle Morphology, and Exploratory Behavior

**DOI:** 10.3389/fneur.2016.00191

**Published:** 2016-11-07

**Authors:** Viythia Katharesan, Martin David Lewis, Robert Vink, Ian Paul Johnson

**Affiliations:** ^1^Anatomy and Pathology, The University of Adelaide, Adelaide, SA, Australia; ^2^Mind and Brain Theme, South Australian Health and Medical Research Institute, Adelaide, SA, Australia; ^3^Health Sciences Divisional Office, University of South Australia, Adelaide, SA, Australia

**Keywords:** motor neurons, cytokines, inflammaging, rat, neurodegeneration, ageing

## Abstract

An overall increase in inflammatory cytokines with age in both the blood and the central nervous system (CNS) has been proposed to explain many aspects of ageing, including decreased motor function and neurodegeneration. This study tests the hypothesis that age-related increases in inflammatory cytokines in the blood and CNS lead to facial motor neuron degeneration. Groups of 3–5 female Sprague-Dawley rats aged 3, 12–18, and 24 months were used. Twelve cytokines interleukin (IL)-1α, IL-β, IL-2, IL-4, IL-5, IL-6, IL-10, IL-12p70, IL-13, tumor necrosis factor-α (TNFα), interferon-γ, and granulocyte macrophage-colony stimulating factor were measured in blood plasma and compared with those in the brainstem after first flushing blood from its vessels. The open-field test was used to measure exploratory behavior, and the morphology of the peripheral target muscle of facial motor neurons quantified. Total numbers of facial motor neurons were determined stereologically in separate groups of 3- and 24-month-old rats. Ageing rats showed a significant 30–42% decrease in blood plasma (peripheral) concentrations of IL-12p70 and TNFα and a significant 43–49% increase in brainstem (central) concentrations of IL-1α, IL-2, IL-4, IL-10, and TNFα. They also showed significant reductions in motor neuron number in the right but not left facial nucleus, reduced exploratory behavior, and increase in peripheral target muscle size. Marginal age-related facial motoneuronal loss occurs in the ageing rat and is characterized by complex changes in the inflammatory signature, rather than a general increase in inflammatory cytokines.

## Introduction

**Figure 1:**
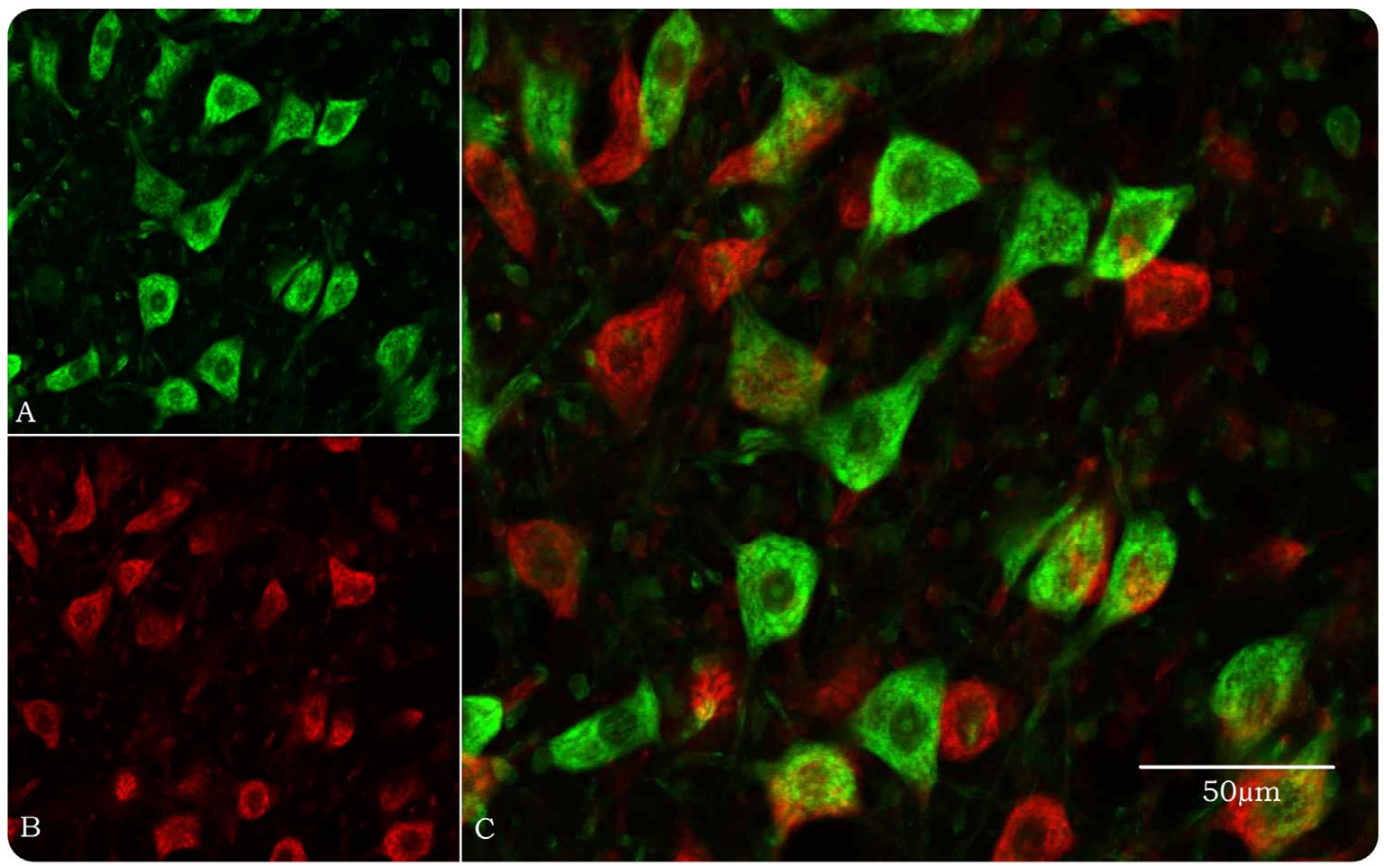
**Optical disector method in the confocal microscope for stereological estimation of total numbers of facial motor neurons**. **(A)** Motor neurons, pseudocolored green, are identified by their size and morphology when their nucleoli, nuclei, and Nissl bodies are fluorescently stained by YOYO-1 iodide. Neuroglial cells are approximately 20% of the size of motor neurons and easily discounted from counting. **(B)** Same field as in **(A)** but with a 10-μm Z-step and pseudocolored red. **(C)** Images **(A)** and **(B)** merged so that only motor neurons in the look-up section (green) are counted.

While there is abundant information from experimental studies on the survival requirements of young motor neurons, not much is known about adult and aged motor neurons, and there is reason to think that young motor neurons are inappropriate models for age-related neurodegeneration, such as MND (1). Using a nerve avulsion model and stereological analysis in the confocal microscope (Figure 1), we previously reported age-related differences in rat facial motor neuron survival (2–6). Here, we consider whether age-related increases in inflammation “inflammaging” (7) can affect facial motor neuron survival, by correlating age-related motoneuronal survival with changes in the central nervous system (CNS) parenchyma in the brainstem at the level of the facial nucleus and in the blood.

Inflammation and its regulation by cytokines is considered to play an important role in both healthy aging of the nervous system and neurodegeneration (7, 8). Inflammatory cytokines have also been reported to affect motor functions (9). The link between aging and inflammation has led to the concept of “inflammaging” (10), which is defined as a low-grade chronic inflammatory state associated with the aging process. This concept centers on age-related inflammatory cytokine-driven innate immune responses in the peripheral immune system. Inflammaging has also been adduced to help explain age-related neuronal degeneration in the CNS (7, 11). The extent to which information derived from studies of the peripheral immune system can be transposed to the CNS however is unclear because there are both unique immunocompetent cells (microglia) in the CNS, and there is evidence of significant cross talk between changes in the peripheral immune system and changes in the aging CNS (12). Almost all cytokines in the peripheral immune system are also produced in the CNS (13), and cytokines produced peripherally can act on the CNS through several mechanisms (14–16). Just how CNS and peripheral cytokines alter with age, however, is unclear as typical CNS samples also contain large amounts of peripheral blood (17, 18), raising the possibility that larger changes in systemic cytokines may mask smaller changes in CNS cytokines. In this study, we have sought to distinguish age-related changes in CNS cytokines from those occurring systemically, by analyzing the brainstem at the level of the facial nucleus after flushing the blood from its vessels. Using a multivariate approach, we have studied 12 cytokine-proteins that are implicated in both aging (7, 8, 19) and neurodegenerative conditions (20, 21) to identify the inflammatory signature characteristic of healthy aging in rats. The cytokines studied were interleukin (IL)-α, IL-β, IL-2, IL-4, IL-5, IL-6, IL-10, IL-12p70, IL-13, tumor necrosis factor-α (TNFα), interferon (IFN)-γ, and granulocyte macrophage-colony stimulating factor (GM-CSF). Of these cytokines, IL-1α, IL-β, IL-2, IL-6, IL-12p70, TNF-α, IFNγ, and GM-CSF are pro-inflammatory, whereas IL-4, IL-5, IL-6, IL-10, and IL-13 are anti-inflammatory (22, 23). Age-related changes in cytokines have been compared with age-related changes in total numbers of facial motor neurons as well as age-related changes in the fiber density of the peripheral target muscle and changes in exploratory behavior.

## Materials and Methods

### Animals

For the cytokine analysis, morphometry of muscle, and open-field tests, groups of 3–5 female Sprague-Dawley rats aged 3, 12–18, and 24 months were used. For motor neuron counts, groups of 6–12 rats aged 3 and 24 months were used. Aging rats are not available commercially in Australia. Rats were therefore obtained as adults and maintained until they are 24-month-old in the rodent facility of the University of Adelaide before use. While the maximum reported lifespan of the *ad libitum*-fed Sprague-Dawley rat is 36 months (24), we found approximately 50% of the rats in our study had died by 24 months, as reported previously for this strain (2, 4, 25). Animals were housed under a standard 12-h on/off lighting regime and given food and water *ad libitum*. The experimental study complied with the Australian code for the care and use of animals for scientific purposes (2016) and was approved by the University of Adelaide Animal Ethics Committee (M-57-2013). The aging animals analyzed here did not have significant health issues although they did have age-related conditions, such obesity, lipomas, and arthritis that are typical of an aging population.

### Stereological Counts of Facial Motor Neurons

In rats terminally anesthetized with sodium pentobarbitone, the brain was fixed by intracardiac perfusion with 4% phosphate-buffered paraformaldehyde following a saline rinse. Also, 100 μm Vibratome sections were cut serially through the facial nucleus and numbers of motor neurons estimated in every fifth section using an optical disector method modified for use in the confocal scanning laser microscope as described previously (3).

### Image Processing and Morphometric Analysis of Peripheral Target Muscle

Also, 5-μm microtome sections of the paraffin-embedded peripheral muscle targets of facial motor neurons (i.e., snout muscle) were stained with hematoxylin and eosin for general muscle morphology measurements (26) using the segmentation and analysis method (FIJI, Adelaide Microscopy). High quality 20× magnification images were collected *via* the NDPview software to ensure the images retained characteristics from initial acquisition for analysis and would not require enhancing in terms of brightness or contrast. Briefly, the scale on the images was set so that all subsequent measurements were correct (e.g., *X* number of pixels for a known distance). The threshold of images was then automatically adjusted by Image J before converting the colored image to a binary image. The binary watershed function was used to ensure real muscle fibers were being segmented as “real particles.” Finally, particles were identified as the output type “maxima within tolerance” and noise tolerance was set to 200.00 before being analyzed.

### Functional Test

An open-field test was used as a measure of exploratory behavior (27). A 100 cm × 100 cm square box acted as the “open field,” and rats were placed in the center of the open-field arena. Movement, in terms of total distance traveled, was then recorded for a period of 5 min. To ensure consistency between groups, the time at which the test was conducted, color, and texture of the open-field box, lighting, temperature, ambient noise, and olfactory cues were all controlled for. The Stoelting “ANY-maze” software was used as the tracking system that automated this functional test (28).

### Cardiac Puncture, Saline-Perfusion, Protein Extraction, and Estimation

Rats were deeply anesthetized by inhalation of 5% isofluorane in 2 l oxygen/min, and while the anesthetic nose cone was still attached, cardiac puncture was performed to withdraw blood into EDTA-coated blood tubes. Blood plasma was retrieved and stored at −80°C. Immediately following cardiac puncture, rats were perfused transcardially with approximately 200 ml of sterile saline until the fluid flowing out of the right atrium was clear. The animals were then decapitated and the brainstem removed, trimmed at the mid pons level and approximately 1 mm below the lower border of the pons to ensure it contained the facial nucleus, snap-frozen, and stored at −80°C. Frozen brainstem samples were homogenized in lysis buffer made up with PBS, triton-X, and protease inhibitors (Roche, cOmplete tablets). The supernatant was retrieved from homogenized samples and stored at −80°C. The BioRad DC Protein Assay (a modified Lowry method) was used to quantify the amount of protein in each sample as per the manufacturer’s instructions.

### Multiplex Assay

Bio-Plex Pro Rat 12 plex cytokine assay kits (BioRad, New South Wales) were used to measure the concentration of 12 cytokines within each sample. Samples were loaded onto 96 well plates in duplicates (3- and 12- to 18-month-old rats) and triplicates (24-month-old rats). Plates were read using a Magpix Luminex multiplexing platform, which uses a fluorescent imager (Abacus ALS, Queensland) and data expressed as picogram/milliliter of concentration. Experimental data were calibrated against standard curves of all 12 cytokines (BioRad, New South Wales). To validate the accuracy of the multiplex assay, a spike recovery analysis was performed. This involved obtaining readings for cytokine standards serially diluted in buffer as per the manufacturer’s instruction and comparing these with readings for cytokines diluted in brain homogenates (“spike recovery”). The later represented the form in which the cytokines were measured in rats of different ages in this study. As seen in Table 1, slope differences of ≤30% were found. Using a Parallelism approach, this is generally taken to indicate that there are minimal effects of the matrix on the assays and resultant standard curves (29).

**Table 1:** **Slope differences (% difference) of 3- and 24-month homogenate samples compared with standard curves**.

Cytokine standard	Sample% difference	
	3 months	24 months
IL-1α	0.59	0.09
IL-1β	8.78	4.07
IL-2	6.42	10.96
IL-4	4.07	−7.56
IL-5	30.51	3.82
IL-6	−7.22	11.56
IL-10	28.07	−2.24
IL-12 (p70)	−8.32	3.57
IL-13	9.67	−1.61
TNF-α	−1.82	−2.46
IFN-γ	−1.01	4.22
GM-CSF	−21.79	−1.09

### Statistical Analysis

Mann–Whitney *U* tests were used for comparisons of motor neuron number, morphometric analysis of muscle, and functional test results. A general linear model (SPSS statistics 22, IBM) was used to generate descriptive statistics for all three age groups and to check for interactions between cytokines. The 12 cytokines within the same sample were treated as “repeated measures” within each animal. Age categories were treated as between-subjects factors, and the 12 cytokines were treated as within-subjects factors. Dunnett’s *post hoc* test was used to test for differences between cytokines. The omnibus/homogeneity test confirmed that the spread of scores was roughly equal across the three age groups, which meant that the comparisons were between populations with equal variances. A multivariate test was then run between age categories to determine the significance of differences within cytokines in the different age groups. Statistical significance of *p* < 0.05, *p* < 0.01, and *p* < 0.001 is reported.

## Results

### Age-Related Changes in Facial Motor Neurons and Their Peripheral Targets

**Figure 2:**
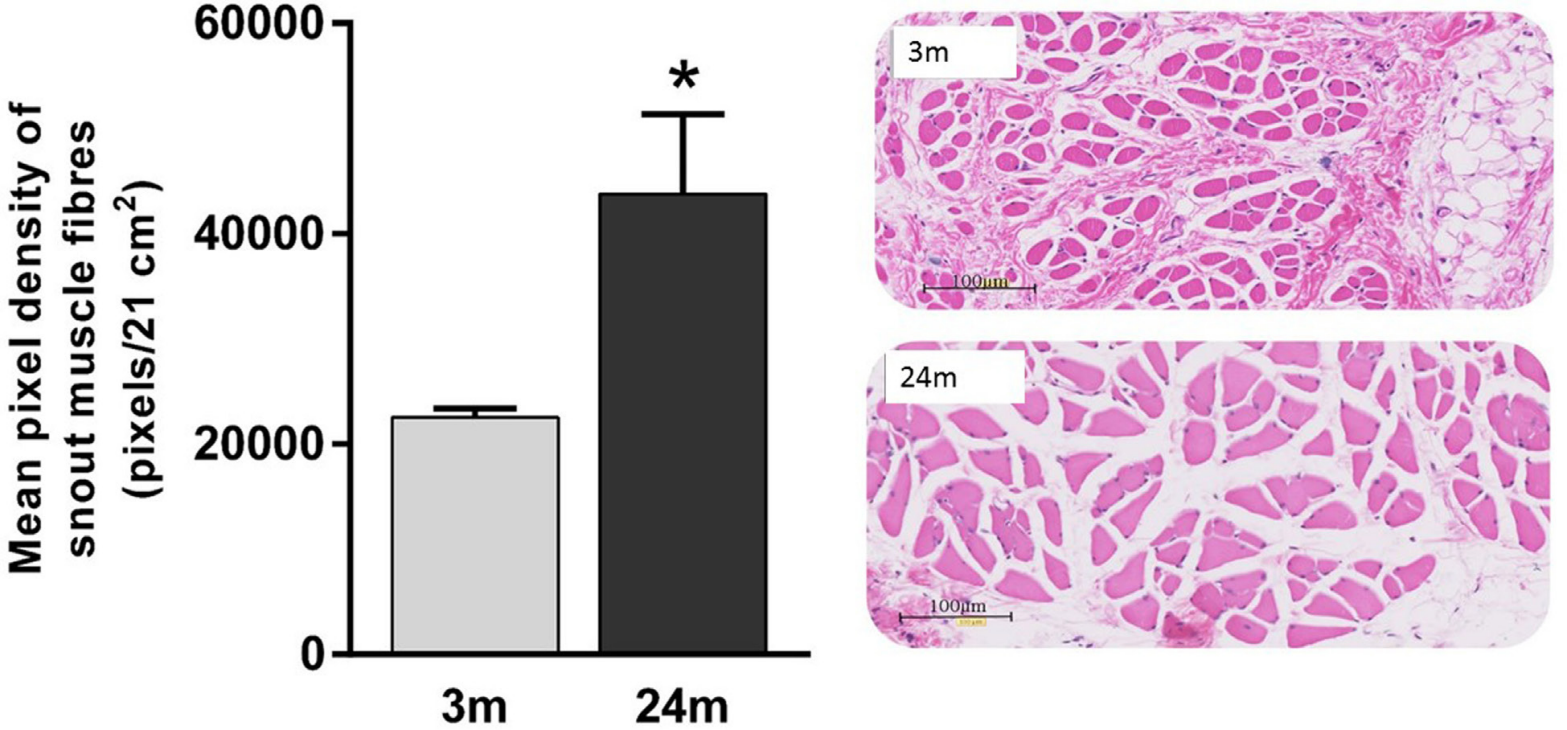
**Significantly lower pixel density measurements (pixels/21 cm^2^) of 3-month-old rats’ snout muscle fibers compared with 24-month-old rats (Mann–Whitney *U* test, *p* < 0.05)**. Representative 20× H&E images of 3-month (gray label, *n* = 5) and 24-month (black label, *n* = 4) images.

Mean total numbers of motor neurons in the brainstems of 24-month-old rats were 22% lower than those of 3-month-old rats (Table 2). While this reduction was statistically significant for the right facial nucleus (24% reduction, *p* = 0.041), it just failed to reach significance for the left (19% reduction, *p* = 0.052). This probably reflects the small (*n* = 6) sample size for the 24-month-old rats. In contrast to the age-related reduction in number of facial motor neurons, analysis of the snout muscle, which represents the peripheral targets of these motor neurons, reveled an increase in muscle fiber size. Thus, mean pixel density measurements (pixels/21 cm^2^) from segmentation and analysis of 20× images showed that 3-month-old rats had 49% smaller (*p* < 0.05) peripheral muscle fiber densities (22,535 ± 822) compared with 24-month-old rats (43,786 ± 7564) (Figure 2).

**Table 2:** **Numbers of motor neurons in the left and right facial nuclei of 3- and 24-month-old rats**.

	3 month-old rats			24 month-old rats		
	Left nucleus	Right nucleus	Left + right nucleus	Left nucleus	Right nucleus	Left + right nucleus
	3404	3676	7080	2518	2953	5471
	3118	3622	6740	2461	3063	5524
	3385	3631	7016	3872	3722	7594
	3233	3666	6899	1982	1842	3824
	3090	3305	6395	1866	1802	3668
	3307	3222	6529	2389	1998	4387
	2958	2306	5264			
	3501	4135	7636			
	2821	3329	6150			
	2232	2295	4527			
	3127	3990	7117			
	3105	3398	6503			
*n*	12	12	12	6	6	6
Mean	3106.75	3381.25	6488.00	2514.67^+^	2563.33*	5078.00
SEM	97.20	165.08	246.85	292.43	324.73	597.82
% loss vs. 3-month-old rats				19.06	24.19	21.73

*p = 0.041.

*^+^p = 0.052 vs. nucleus of same side of 3-month-old rats*.

### Age-Related Changes in Open-Field Exploratory Behavior

**Figure 3:**
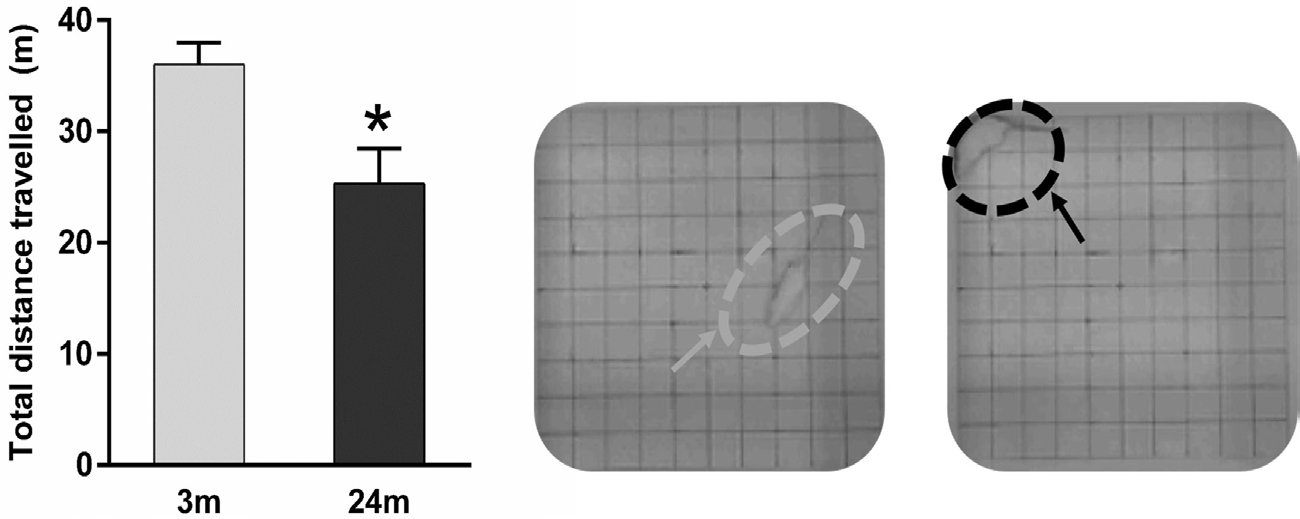
**Mean ± SEM (meter) total distance traveled in the open field**. Asterisk denotes *p* < 0.05 (Mann–Whitney *U* test) showing 24-month-old rats (*n* = 5) traveling a significantly shorter distance vs. 3-month-old rats (*n* = 4). Example representative images of an adult rat exploring the open-field (gray arrow) and an aged rat crouched in the open-field corner (black arrow).

In general, 3-month-old rats were more active. This qualitative observation was confirmed using total distance traveled (meters) during the open-field test, where 3-month-old rats showed 44% more (*p* < 0.05) exploratory behavior than 24-month-old rats (Figure 3).

### Age-Related Changes in Brainstem and Serum Cytokine Levels

**Figure 4:**
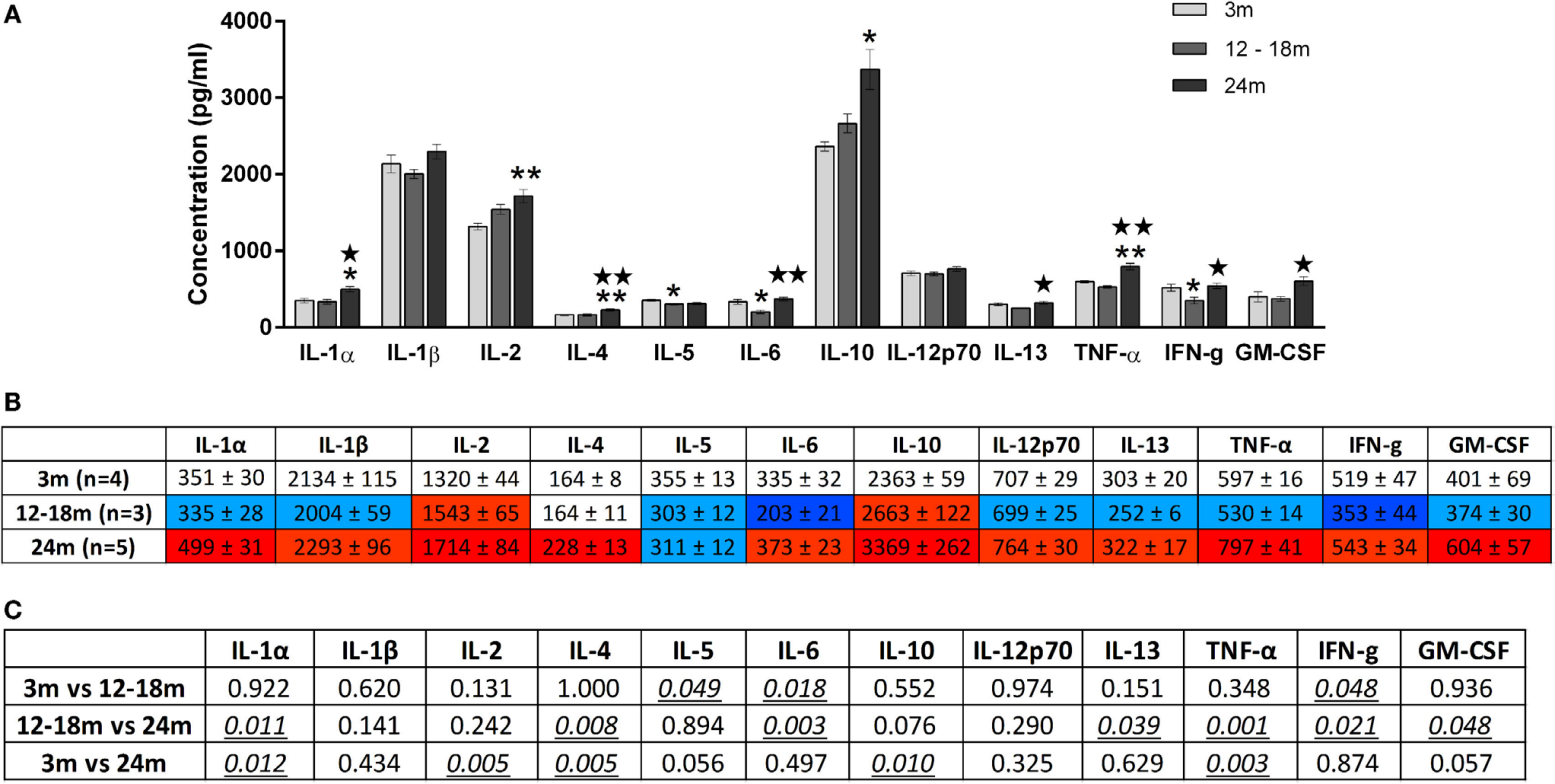
**(A)** Cytokine levels (picogram/milliliter, mean ± SEM) in the brainstem of 3-, 12- to 18-, and 24-month-old rats. A single asterisk and double asterisks denote *p* < 0.05 and *p* < 0.01 vs. 3 months, respectively. A single star and double stars denote *p* < 0.05 and *p* < 0.01 vs. 12–18 months, respectively. **(B)** Color-coded table showing changes in cytokine levels in the brainstem (mean ± SEM). Compared to 3-month-old rats (no color), >15% increase is represented by light red and >25% by dark red, >15% decrease is represented by light blue and >25% by dark blue. **(C)** Summary of *p*-values of brainstem cytokine changes with age. Statistically significant differences are italicized and underlined. Note that changes in IL-5 and GM-CSF showed trends that were not statistically significant.

**Figure 5:**
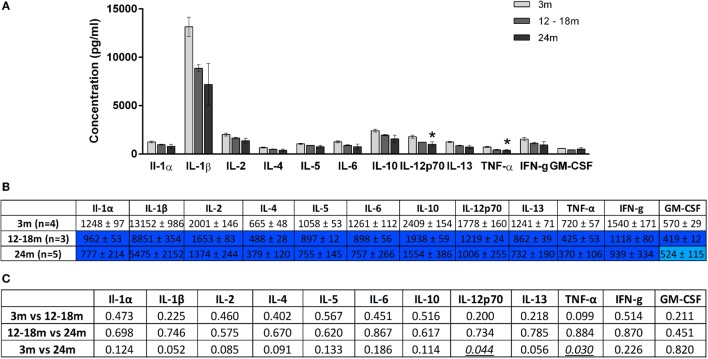
**(A)** Cytokine levels (picogram/milliliter, mean ± SEM) in the plasma of 3-, 12- to 18-, and 24-month-old rats. Asterisk denotes *p* < 0.05 vs. 3 months. **(B)** Color-coded table showing changes in cytokine levels in the plasma (mean ± SEM). Compared to 3-month-old rats (no color), >15% decrease is represented by light blue and >25% by dark blue. **(C)** Summary of *p*-values of plasma cytokine changes with age. Statistically significant differences are italicized and underlined. Note that changes in IL-1β and IL-13 showed trends that were not statistically significant.

Overall, increasing age from 3 to 24 months was associated with an increase in brainstem cytokine levels (Figure 4) and a decrease in plasma cytokine levels (Figure 5). Analysis of rats aged 12–18 months revealed that this did not affect cytokines uniformly. Thus, in the brainstem of 12- to 18-month-old rats, the concentrations of three cytokines were significantly lowered by 17–65% compared to 3-month-old rats (*p* < 0.05). The cytokines and their concentrations (picogram/milliliter) were IL-5 (303 ± 12 vs. 355 ± 13), IL-6 (203 ± 21 vs. 335 ± 32), and IFNγ (353 ± 44 vs. 519 ± 47). In contrast, there were no significant differences in plasma samples of 3- and 12- to 18-month-old rats. This suggests that regional differences (plasma vs. brainstem) in age-related changes in cytokines are in place by 12–18 months of age.

In the brainstem of 24-month-old rats, the concentrations of five cytokines were significantly increased by 23–30% compared to 3-month-old rats. The cytokines and their concentrations (picogram/milliliter) were IL-1α (499 ± 31 vs. 351 ± 30, *p* < 0.05), IL-2 (1714 ± 84 vs. 1320 ± 44, *p* < 0.01), IL-4 (228 ± 13 vs. 164 ± 8, *p* < 0.01), IL-10 (3369 ± 262 vs. 2363 ± 59, *p* < 0.05), and TNFα (797 ± 41 vs. 597 ± 16, *p* < 0.01). While the three cytokines whose concentrations were decreased in the brainstems of 12- to 18-month-old rats were no longer decreased at 24 months, they did not contribute to the increase in cytokines seen at 24 months. GM-CSF was also higher in the 24-month-old group although this just failed to reach statistical significance. When compared to 12- to 18-month-old rats, the concentrations of seven cytokines were significantly increased by 28–61% in the brainstem of 24-month-old rats. The cytokines and their concentrations (picogram/milliliter) were IL-1α (335 ± 28 vs. 499 ± 31, *p* < 0.05), IL-4 (164 ± 11 vs. 228 ± 13, *p* < 0.01), IL-6 (203 ± 21 vs. 373 ± 23, *p* < 0.01), IL-13 (252 ± 6 vs. 322 ± 17, *p* < 0.05), TNF-α (530 ± 14 vs. 797 ± 41, *p* < 0.01), IFNγ (353 ± 44 vs. 543 ± 34, *p* < 0.05), and GM-CSF (374 ± 30 vs. 604 ± 57, *p* < 0.05). Three of these cytokines (IL-1α, IL-4, and TNF-α) were also elevated in 24-month-old rats when compared with 3-month-old rats, indicating that changes occur earlier and are longer lasting in these cytokines compared to other brainstem cytokines.

A contrasting picture emerged for the effects of aging on cytokine levels in plasma derived from peripheral blood. Compared to 3-month-old rats, 24-month-old rats had significantly lower mean plasma concentrations (picogram/milliliter) of IL-12p70 (1006 ± 255 vs. 1778 ± 160) and TNF-α (370 ± 106 vs. 720 ± 57) (Figure 5). Interestingly, while TNF-α was decreased in the plasma of 24-month-old rats, it was increased in the brainstem. Serum concentrations of IL-1β and IL-13 were also lower in 24-month-old rats, although this just missed statistical significance. In contrast to the significant increases in brainstem cytokines seen in 24-month-old rats compared with 12- to 18-month-old rats, no significant differences in plasma cytokine concentrations were found when these two age groups were compared. Thus, changes in plasma cytokine concentrations appear to have stabilized by 12–18 months, whereas changes in brainstem cytokine concentrations continue up to 24 months.

## Discussion

We report that aging (24-month-old) rats (i) have lower blood plasma (peripheral) inflammatory markers, (ii) have higher brainstem (central) inflammatory markers, (iii) show reduced exploratory behavior, (iv) have larger peripheral target muscle for facial motor neurons, and (v) have fewer facial motor neurons than 3-month-old rats. This points to complicated changes occurring in both the inflammatory signature and peripheral target interactions of aging rats that are associated with age-related motor neuron loss.

The median lifespan of *ad libitum*-fed Sprague-Dawley rats is 24–34 months (30, 31) and in line with other studies, we found that 50% of our *ad libitum*-fed Sprague-Dawley rats died by the age of 24 months (4, 25). It is possible that the age-related health changes found in the rats analyzed here such as obesity, lipomas, and arthritis have contributed to the changes in inflammatory markers measured. However, these changes, often referred to as “frailties” (32, 33), are commonly found with age and so to have used rats where such age-related conditions are absent may not have been representative of normal aging. It is also possible that rats showing reduced mortality at 24, such as Fischer 344 rats and diet-restricted rats (2, 4), may show different cytokine changes.

We found statistically significant increases in IL-1α, IL-2, IL-4, IL-6, IL-10, IL-13, TNF-α, IFNγ, and GM-CSF in the brainstem. Of these, IL-1α, IL-2, TNF-α, IFNγ, and GM-CSF are pro-inflammatory, IL-4, IL-10, and IL-13 are considered anti-inflammatory, and IL-6 falls under both categories. We also found statistically significant decreases of IL-12p70 and TNF-α in the plasma, both of which are pro-inflammatory. While this is a complicated picture, our results could be taken to indicate that aging is associated with a general decline in peripheral inflammatory cytokines and a general increase in central inflammatory cytokines. Age-related increases in CNS cytokines have been reported by others (12, 34–36) and associated with increased vulnerability of the CNS to injury (37–39) as well as implicated in the development of age-related neurodegeneration (40–42). In contrast to the brainstem region of the CNS, we find that peripheral cytokines show a general decline with aging, which is at odds with initial studies on “inflammaging” by others showing increased levels of individual inflammatory markers in the periphery, especially IL-6 (43–45). Notwithstanding the difficulty of extrapolating from rats to human lifespan, one possible reason for this difference could be that early studies employed individual ELISA kits/antibodies each with different sensitivities for single cytokines and used different samples with each kit. Also, the view that peripheral cytokines increase with advancing age has been largely based on measurements of IL-6 even though Franceschi (46) showed that other cytokines, such as GM-CSF, were reduced in healthy aged humans. This highlights the need to study many inflammatory mediators within the same sample.

In adult mice, elevated levels of peripheral inflammatory cytokines after LPS challenge also decrease exploratory behavior and is believed to facilitate recovery from acute infections (47, 48). In our study, we found lower levels of exploratory behavior in 24-month-old rats, but this was associated instead with lower levels of peripheral cytokines compared to 3-month-old rats. Only in the brainstems of 24-month-old rats did we find evidence of an increase in inflammatory cytokines. These results indicate that age, the site of inflammation (central or peripheral), and its time-course (acute or chronic) all likely contribute to behavioral changes.

Peripheral muscle wasting has been associated with increased cytokine signaling, especially with TNF-α largely implicated in the process (49, 50). The larger peripheral target muscle morphometry of 24-month-old rats observed in this study, compared to 3-month-old animals could be associated with the reduced levels of peripheral inflammatory cytokines noted. A link between peripheral target size and motor neuron survival is well known from studies of the developing nervous system (51, 52). This has generally been linked to the ability of the peripheral target to provide neurotrophic support to developing motor neurons (53, 54). In our study of aging rats, an increase in peripheral target size correlated with a decrease in motor neurons. Whether this reflects a decrease in peripheral neurotrophic support with age or is simply a result of the generally larger size of the aging rats is unknown.

The initial concept of inflammaging revolved around the low-grade amplification of pro-inflammatory cytokines (55, 56). However, more recent studies show increases in both pro- and anti-inflammatory cytokines with advancing age (57). This more complicated pattern of change with aging, is consistent with our current results, indicating that aging is not a simple matter of increased inflammation in the whole animal. Acknowledging that only 50% of the rats in our study survived to 24 months, and so must be considered aging “survivors,” the general decrease in peripheral cytokines could be viewed beneficial, possibly ameliorating age-related increases in other inflammatory mediators that were not measured here. Further studies, perhaps employing heterochronic parabiosis, involving the surgical attachment of young and old organisms so that they share a common vascular system (58, 59) are needed to address this point. Neither do we know if the rats that reached 20 months but failed to reach 24 months of age showed increased levels of peripheral cytokines, in keeping with the orthodox concept of inflammaging (60), since we did not analyze the blood in these rats that died early. Our data show no consistent increase in most of the cytokines measured from 3 to 12–18 to 24 months, but we cannot discount changes occurring around the 24-month mark in these cytokines. While we have only looked at 12 cytokines, this result forces the conclusion that changes in the total inflammatory signature are likely to characterize aging and that these changes are different in the CNS and periphery. Notwithstanding the possibility that our results on the brainstem may not generalize the rest of the CNS, this view runs contrary to the concept of “inflammaging” for the whole animal and the various ways this concept has been adduced to explain age-related neuronal degeneration (48, 61).

## Conclusion

We show that the peripheral innate immune system of adult rats has higher levels of cytokines than the brainstem, and this balance is reversed in aging rats. We also show that inflammatory changes in the aging brainstem are different to those occurring in the blood. If our results for the brainstem at the level of the facial nucleus can be confirmed generally for the CNS, they may have implications for the design of therapeutic strategies for age-related diseases affecting the CNS or other parts of the body. The discrepancy between our results and others calls into question the importance of changes of cytokines with age. Our multi-analyte approach leads us to suggest that changes in all inflammatory mediators, or the “inflammatory signature,” may better link inflammation with regional aging processes. The changes in the inflammatory signature may involve an intrinsic loss in competency of peripheral immunity with age or be the result of a regulatory system where increased cytokine levels in the CNS feedbacks to the periphery.

## Author Contributions

VK – experimental design, data collection, analysis and interpretation, wrote manuscript. ML, RV, and IJ – experimental design, supervised development of work, manuscript evaluation.

## Conflict of Interest Statement

The authors declare that the research was conducted in the absence of any commercial or financial relationships that could be construed as a potential conflict of interest.
